# Synthesis and Biological Evaluation of Triazolyl 13α-Estrone–Nucleoside Bioconjugates

**DOI:** 10.3390/molecules21091212

**Published:** 2016-09-10

**Authors:** Brigitta Bodnár, Erzsébet Mernyák, János Wölfling, Gyula Schneider, Bianka Edina Herman, Mihály Szécsi, Izabella Sinka, István Zupkó, Zoltán Kupihár, Lajos Kovács

**Affiliations:** 1 Department of Medicinal Chemistry, University of Szeged, Dóm tér 8, H-6720 Szeged, Hungary; bodnar.brigitta@med.u-szeged.hu; 2 Department of Organic Chemistry, University of Szeged, Dóm tér 8, H-6720 Szeged, Hungary; bobe@chem.u-szeged.hu (E.M.); wolfling@chem.u-szeged.hu (J.W.); schneider@chem.u-szeged.hu (G.S.); 3 1st Department of Medicine, University of Szeged, Korányi fasor 8-10, H-6720 Szeged, Hungary; herman.bius@gmail.com (B.E.H.); szecsi.mihaly@med.u-szeged.hu (M.S.); 4 Department of Pharmacodynamics and Biopharmacy, University of Szeged, Eötvös u. 6, H-6720 Szeged, Hungary; sinkaiza@gmail.com (I.S.); zupko@pharm.u-szeged.hu (I.Z.)

**Keywords:** nucleosides, 13α-estrone, copper-catalyzed alkyne–azide click reaction, triazoles, antiproliferative, 17β-HSD1

## Abstract

2′-Deoxynucleoside conjugates of 13α-estrone were synthesized by applying the copper-catalyzed alkyne–azide click reaction (CuAAC). For the introduction of the azido group the 5′-position of the nucleosides and a propargyl ether functional group on the 3-hydroxy group of 13α-estrone were chosen. The best yields were realized in our hands when the 3′-hydroxy groups of the nucleosides were protected by acetyl groups and the 5′-hydroxy groups were modified by the tosyl–azide exchange method. The commonly used conditions for click reaction between the protected-5′-azidonucleosides and the steroid alkyne was slightly modified by using 1.5 equivalent of Cu(I) catalyst. All the prepared conjugates were evaluated in vitro by means of MTT assays for antiproliferative activity against a panel of human adherent cell lines (HeLa, MCF-7 and A2780) and the potential inhibitory activity of the new conjugates on human 17β-hydroxysteroid dehydrogenase 1 (17β-HSD1) was investigated via in vitro radiosubstrate incubation. Some protected conjugates displayed moderate antiproliferative properties against a panel of human adherent cancer cell lines (the protected cytidine conjugate proved to be the most potent with IC_50_ value of 9 μM). The thymidine conjugate displayed considerable 17β-HSD1 inhibitory activity (IC_50_ = 19 μM).

## 1. Introduction

**Figure 1:**
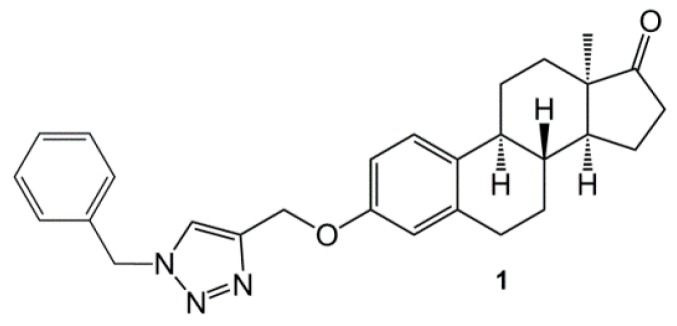
Structure of 3-*O*-[(1-benzyl-1*H*-1,2,3-triazol-4-yl)methyl]-13α-estrone.

Estrogens are synthesized biochemically in a multistep process from cholesterol in human body [1], and are responsible for the development of secondary sexual characteristics in females and maintenance of central nervous system, cardiovascular system and bones. Since they play a crucial role in the cell proliferation, overproduction of estrogens may lead to enhanced proliferation of hormone sensitive cells, resulting in hormone dependent cancers: ovarian, uterine, breast, prostate and endometrial [2]. Estrone-based anticancer drugs have been developed as antiproliferative/antihormonal or cytotoxic agents acting on non-hormonal targets [3]. Antiproliferative estrogens exert their activity as enzyme inhibitors or as antiestrogens (acting through their receptors). Although the proliferation process is rather complex, one of potential enzymes which plays crucial role in the proliferation process of some cancer cell types is the human 17β-hydroxysteroid dehydrogenase 1 (17β-HSD1). It catalyzes the reduction of estrone to 17β-estradiol, which enhances the proliferation of certain cancer cells [4]. High activity of this isozyme can be detected in female reproductive tissues, e.g., in the ovaries and in the placenta [5]. 17β-HSD1 has been found to be responsible for the local overproduction of 17β-estradiol in various breast cancers and ovarian cancers [6]. The inhibition of 17β-HSD1 with suitable pharmacons decreases synthesis of 17β-estradiol and causes significant estrogen deprivation and antitumor effect in hormone dependent cancers, therefore 17β-HSD1 inhibitors could be promising candidates of anti-estrogen therapy [7,8]. Regardless of the mechanism of antiproliferative action, there is a general requirement in the development of all estrone-based anticancer drugs: the lack of estrogenic activity. Chemical modifications of estrone may lead to compounds lacking hormonal behavior [3,9,10]. Substitution at C-2, opening of ring D or inversion at C-13 of the estrane skeleton usually leads to the loss of estrogenic activity [11,12,13,14,15,16]. 13α-Estrone may be an excellent scaffold for the design of hormonally inactive agents having antiproliferative activity and fortunately it is readily available from native 13β-estrone by the method of Yaremenko and Khvat, using 1,2-phenylenediamine and acetic acid [17]. We recently published 13α-estrone, 1,2,3-triazolyl 13α- and d-secoestrone derivatives possessing substantial cytostatic and/or 17β-HSD1 inhibitory properties [18,19,20,21,22]. 13α-Estrone itself exerted outstanding 17β-HSD1 inhibitory activity with an IC_50_ value comparable to that of the reference estrone [22]. Concerning the triazoles, the heterocyclic ring was introduced to C-3 or to C-16 directly or via a short linker. The cell growth-inhibitory potential depended on the position of the triazolyl moiety and on the nature of the functional group at C-3. 3-Hydroxy or 3-ether derivatives displayed lower cytostatic potentials than their 3-*O*-[(1-benzyl-1*H*-1,2,3-triazol-4-yl)methyl] counterparts. The latter triazoles displayed one order of magnitude higher activities (submicromolar IC_50_ values) than the earlier described potent 16-triazolyl 3-*O*-benzyl ethers [18]. It can be stated that concerning the antiproliferative behaviour of triazolyl 13α-estrones, functionalization at C-3 over C-16 seems more preferential. One of the most potent antiproliferative compounds was 3-*O*-[(1-benzyl-1*H*-1,2,3-triazol-4-yl)methyl]-13α-estrone bearing intact ring D (**1**, Figure 1). Based on its remarkable cytostatic potential (IC_50_ = 0.3–0.9 μM, [20], this 13α-estrone triazole conjugate (**1**) may be used as a model compound for further derivatization with dual aim: to improve its cell growth-inhibitory potential to nanomolar scale, and to enhance its tumor selectivity. A potential route to enhance this selectivity is to prepare bioconjugates. Our recent results suggest that the triazole ring at 3-*O* should remain to retain the biological activity, but the azide counterpart used in the CuAAC reaction may be another biomolecule, such as a nucleoside.

The advantage of using a nucleoside azide could be either the higher activity on targeted enzymes overrepresented in cancer cells or an enhanced cellular uptake of the bioconjugates in these cells compared to the healthy ones. As the cancer cells require higher amount of nucleoside building blocks for their proliferation, they have significantly higher uptake of nucleosides by the different nucleoside transporters [23,24,25]. The nucleoside–steroid bioconjugates, which have been synthesized so far for anticancer purposes contain the nucleoside units as the bioactive components, like 2′-deoxy-5-fluorouridine or coenzyme mimics in bisubstrate inhibitors [26,27]. However, to our knowledge nucleosides, which can selectively enhance the transport into the cancer cells, are not used as targeting carriers.

Therefore our aim was to test this possibility in case of an estrone derivative with established antiproliferative activity. If the coupled nucleoside unit increases the active transport of the steroid molecule without effecting the antiproliferative activity of the steroid part, one should see an increased inhibition of cell growth.

The connection types applied in the nucleoside–steroid conjugates prepared so far are generally ester bonds [26,28,29,30,31]. On the other hand, in case of conjugates of polyfunctional biomolecules, the CuAAC method is a widely used alternative [32,33] which forms a stable 1,2,3-triazole ring. 1,2,3-Triazoles (for sake of simplicity hereinafter referred to as “triazoles”; systematic compound names are given in the Materials and Methods section) are extensively used linkers in synthetic bioactive conjugates because of their stability against metabolic degradation and their favourable hydrogen-bonding properties. Incorporation of a triazole ring into the estrane skeleton has additional advantages: it may enhance the water solubility, bioavailability and as mentioned above this structural moiety enhanced the antiproliferative activity of an estrone derivative [18]. The CuAAC is a highly selective coupling method [34] which requires an azide and an alkyne function on the biomolecules and copper(I) ion as a catalyst.

**Scheme 1:**
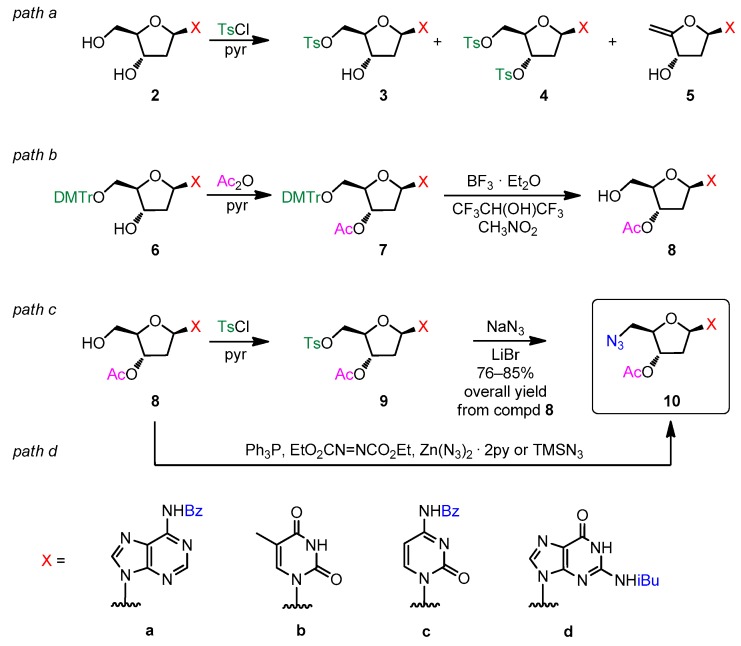
Synthesis of 5′-azido-2′,5′-dideoxynucleosides.

In our case two potential approaches can be considered for the introduction of alkyne and azide moieties suitable for CuAAC reaction: (1) the nucleosides contain the azido group and the steroid has a terminal alkyne function or (2) the nucleosides have the terminal alkyne functional group and the steroid contains the azide. The formation of an alkynyl ether function on the phenolic OH group of an estrone derivative is not problematic, but the derivatization of nucleosides is not as straightforward as it seems. Regarding the first possibility, the preparation of 5′-azido-5′-deoxy nucleosides in the commonly used two-step method (5′-*O*-activation by introduction of tosylate [35,36,37,38,39], mesylate [38], halogen [35,39,40,41,42,43,44,45,46] or other leaving group [47,48] followed by azide substitution is hampered by several side-reactions and variable yields. The most relevant side-reactions are 3′,5′-*bis*-*O*-tosylation, 3′,5′-diazide formation [35], 4′,5′-elimination and 2,3′/5′-anhydronucleoside formation (in the case of pyrimidine nucleosides) [43,47]. As a rule, the yields of these transformations are highly dependent on the identity of nucleobase (cytidine and especially guanine are troublesome) [37,41,42,43,45,47], configuration of sugar moiety [35,49], protecting group pattern [36] of the nucleobase and the sugar, steric congestion [40,44] and the actual method used [39,46,48]. Hence, the overall yields of these reactions are usually not very high, generally around 40%–60% or even lower (Scheme 1, *path a*).

Alternatively, Mitsunobu reaction [50,51,52] with hydrogen azide, trimethylsilyl azide or zinc azide-pyridine complex [53] is also a possible alternative to prepare azides from nucleosides, provided that the senstive sugar moieties survive these conditions (Scheme 1, *path d*).

The second possibility (alkynylation of nucleosides) is also problematic: low yields [54,55,56,57,58], concomintant *O*,*N*-dialkylation hamper the effective derivatization of nucleosides [57,58]; it was also observed that cation chelation and solvents greatly influence the outcome of the reactions [59].

Here we chose the above mentioned 13α-estrone as a starting compound with the aim of synthesizing nucleoside bioconjugates and we planned to investigate the antiproliferative properties and potential 17β-HSD1 inhibitory effect of these conjugates. Based on our earlier experience in the preparation and in vitro biological assays of triazolyl 13α-estrone derivatives with anticancer activity, we have opted for the 3-hydroxy group of 13α-estrone for the introduction of the terminal alkyne function. On the nucleosides the 5′-hydroxy seems to be the best choice because its chemical reactivity is higher than that of 3′, and also the better biocompatibility, as the nucleosides in the cells are mainly derivatized on their 5′-end. Hence, it is more likely that the recognition of the nucleosides on the receptor, enzyme and transporter proteins are favoured in case the nucleosides are only modified on their 5′-end and the rest of the nucleosides are freely available for the proteins.

## 2. Results and Discussion

### 2.1. Preparation of 5′-Azido-2′,5′-dideoxynucleosides

For the preparation of 5′-azido-2′,5′-dideoxynucleosides, first we have followed the tosyl–azide replacement method based on the literature but the isolated yields were significantly lower in our hands (Scheme 1, *path a*) compared to those described in the literature [35,36,37,38,39] therefore we decided to protect the 3′-hydroxy groups (Scheme 1, *path b*). Although the 3′-protection requires two more steps (3′-*O*-acylation and 5′-*O*-deprotection when the starting material is a 5′-*O*-(4,4′-dimethoxytrityl (DMTr))-*N*-acyl-protected nucleoside), it helps avoid the bis-3′,5′-*O*-tosylation and increases the solubility of the nucleosides which might also be a reason of the low yields. We have chosen the acetyl-protection of the 3′-hydroxy because it was considered to be compatible with the final deprotection of the steroid–nucleoside conjugates. The crude acetylated material was used for the 5′-*O*-DMTr deprotection without chromatographic purification. As a commonly used 3% trichloroacetic acid/dichloromethane deprotection resulted in a considerable amount of depurination side-products (mainly in the case of 2′-deoxyadenosine) therefore we have changed the reagent to a deprotection mixture containing the Lewis acid boron trifluoride in a 1,1,1,3,3,3-hexafluoroisopropanol–nitromethane solution [60] (Scheme 1, *path b*).

Using the 5′-OH containing, 3′-*O*-acetyl-protected nucleosides (**8a**–**d**) we have carried out the 5′-*O*-tosylation in pyridine at room temperature and after purification the tosyl–azide exchange reaction in DMF at 50 °C (Scheme 1, *path c*). We have obtained better yields compared to the ones without 3′-*O*-protection but the yields were still not too high, around 50%. Moreover, in case of 2′-deoxguanosine, the tosylation reaction gave a very low yield (<10%), probably due to the very poor solubility. Therefore we stopped our attempts to obtain the tosylate **9d** and its further derivatization was also abandoned.

As we were not satisfied with the isolated yields of azidonucleosides we attempted to improve them by applying Mitsunobu reaction to obtain the 5′-azides directly in a one-step reaction from the 3′-*O*-acetyl-protected nucleosides (Scheme 1, *path d*). The Mitsunobu reaction requires an acid component which should be HN_3_ in our case but we did not want to use a Brønsted acid to avoid the potential glycoside bond cleavage therefore, instead of the protic acid, we have tried two Lewis acids, Zn(N_3_)_2_ 2 py or trimethylsilyl azide which were also used for Mitsunobu reactions [50,51,52,53]. Although we have tried to optimize the reaction conditions by varying the starting materials (2′-deoxyadenosine, thymidine, 2′-deoxycitidine, 2′-deoxyguanosine,), the azodicarboxylate reagents (diethyl or diisopropyl esters), the azide-containing acids and also applying different temperatures (0 °C and room temperature), we were unable to detect a considerable amount of 5′-azido-2′,5′-dideoxynucleosides. Only in case of 2′-deoxyadenosine we have got a 20% of the desired product, by using trimethylsilyl azide reagent at 0 °C in a 1 h reaction time. In all other cases only the 5′-*O*-trimethylsilylated nucleoside side-products were found by mass spectrometry analyses of the newly appearing TLC spots. These side-products have decomposed during the work up procedure giving back the starting nucleosides. As the Mitsunobu reaction failed to produce the desired 5′-azido-2′,5′-dideoxynucleosides, we eventually have used the 3′-*O*-protected–tosyl–azide exchange route to prepare the required amounts of 5′-azido-2′,5′-dideoxynucleosides **10a**–**c** for the conjugation reactions from crude tosylates **9a**–**c** (Scheme 1, *path c*).

### 2.2. Optimization of the Click Reaction between 5′-Azido-nucleoside Derivatives and 3-O-Propargyl-13α-estrone

**Scheme 2:**
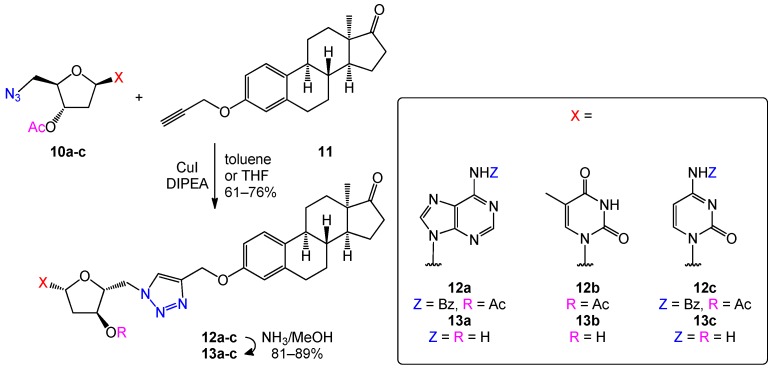
CuAAC conjugation reaction of 3′-*O*-acetyl-5′-azido-2′,5′-dideoxynucleosides and 3-*O*-propargyl-13α-estrone.

The 5′-azido-2′,5′-dideoxynucleosides **10a**–**c** were connected to 3-*O*-propargyl-13α-estrone (**11**) [20] in a CuAAC reaction (Scheme 2). The solvent of the click-reaction was toluene (thymidine and adenosine) or anhydrous tetrahydrofuran (cytidine) due to solubility problems. Initially, we have followed the commonly used literature method (catalytic amounts of copper(II) salts in the presence of sodium ascorbate, aq. *tert*-butanol at room temperature) [32] but to no avail (Table 1). Alternative methods [36,38,61], using 0.01-0.2 equivalent of copper(I) iodide catalyst along with 0.2 equivalent of triphenylphosphane and DIPEA have also been tested but the reaction did not proceed well even if higher temperature, different solvents and prolonged reaction times (3 days) were applied. The highest reaction temperature was limited to as high as 50 °C, to avoid the potential side-reactions on the nucleoside part. We supposed that the reason of the very low yields (<30% according to TLC monitoring) could be the high complex-forming affinity of the *N*-acyl-protected nucleosides which trapped the Cu(I) ion catalysts [54]. Application of inert argone athmosphere did not improve the yield. Therefore we increased the amount of the Cu(I) catalyst and DIPEA to 1.5 equivalent and eliminated triphenylphosphane from the reaction mixture. With this modified method all conjugation reactions were complete in one day at 50 °C according to TLC and the final isolated yields of protected conjugates **12a**–**c** were acceptable (Table 1).

### 2.3. Optimization of the Deprotection of Conjugates (Nucleobase N-Benzoyl and 2′-Deoxy-D-ribose-3′-O-acetyl Deprotection)

The synthesized bioconjugates contained the 3′-*O*-acetyl and *N*-benzoyl protecting groups on the nucleoside moiety which helped increase the solubility in the synthetic reactions but for the biological experiments we needed the unprotected nucleoside conjugates. Aqueous ammonia is commonly used for the deprotection of these protecting groups in the nucleic acid chemistry but the protected conjugates were not soluble in the aqueous media therefore this deprotection method failed. We have tested the Zemplén deacetylation protocol using 0.1 M of sodium methylate in methanol but only the acetyl group was removed from the 3′-hydroxy group. Finally, 4 M ammonia solution in methanol was used at 50 °C which removed all the acyl protecting groups of the conjugates in 16 h to yield derivatives **13a**–**c**.

### 2.4. Antiproliferative Activities

The antiproliferative properties of the newly synthesized nucleoside conjugates were characterized in vitro on a panel of human adherent cancer cell lines (HeLa, MCF-7 and A2780) by means of MTT assays. Structurally similar estrone analogs earlier exhibited growth inhibitory activity against these cells [9,18,19,20,21]. The influence of the nature of the protected or unprotected nucleoside moiety on the cytostatic properties was investigated and the results are shown in Table 2. The protected cytidine conjugate **12c** proved to be the most potent with IC_50_ values in the range 9.0–10.4 μM, although this value is an order of magnitude higher than the value of the 13α-estrone triazole **1** [20]. The removal of the benzoyl and/or acetyl protecting groups from the nucleoside–13α-estrone conjugates **12a**–**c** resulted in unprotected conjugates **13a**–**c** with generally reduced cytostatic properties. Although we do not know the mechanism of action of our conjugates, but compared to the reference steroid **1**, the non-polar benzyl group was replaced by the polar deoxynucleoside units, therefore the lower activity could be due to the more pronounced steric effect and polar properties of the nucleoside units. Interestingly, the conjugates containing less polar but larger protected nucleosides gave higher antiproliferative activity than the unprotected, more polar but smaller nucleoside-containing ones. This fact highlights the importance of both the limited size and non-polar characteristics of the group at C-3 triazolyl moiety of 13α-estrone. On the other hand, this finding also suggests that the hypothesized, potentially selective increase in the uptake of nucleoside conjugates of the estrone derivative might not be operative. The reason of the lower overall uptake could be the decrease of the passive transport of the more polar, nucleoside–estrone derivative through the cell membrane. If the passive transport has much higher contribution to the overall uptake than the nucleoside transporter mediated routes, then the loss of antiproliferative activity of the more polar, unprotected nucleoside–13α-estrone conjugates can be explained by lower concentration of the conjugates inside the cell. Either explanation is true, unfortunately the triazolyl-deoxynucleoside modification of 13α-estrone on C-3 position did not help improving the antiproliferative activity of the model compound **1**.

### 2.5. Inhibition of 17β-HSD1

The newly synthesized nucleoside conjugates **12a**–**c** and **13a**–**c** of 13α-estrone were tested against the human 17β-HSD1 in vitro. The results in Table 3 show that only the unprotected thymidine conjugate **13b** exerted any substantial inhibition, with an IC_50_ value of 19 μM. Other tested conjugates displayed weak inhibition. Compared to our previous results on 13α-estrone and its 3-methyl ether, these conjugates showed lower inhibitory effect, similar to those of the 3-benzyl ether of 13α-estrone [22]. The decrease in the inhibitory potential of nucleoside conjugates is probably caused by the steric effect of the larger nucleoside–triazolyl unit on the C-3 position.

## 3. Materials and Methods 

### 3.1. Chemistry

Melting points (mp) were determined on an IA8103 apparatus (Electrothermal, Stone, UK) and are uncorrected. The reactions were monitored by TLC on Kieselgel-G (Si 254 F, Merck, Darmstadt, Germany) layers (0.25 mm thick); solvent systems (ss): (A) EtOAc, (B) EtOAc/methanol (9:1 *v*/*v*), (C) toluene/isopropanol (1:1 *v*/*v*). The conjugates were detected by spraying with 5% phosphomolybdic acid in 50% aqueous phosphoric acid. For the identification of azides the TLC was placed in a 10% solution of triphenylphosphane in CH_2_Cl_2_ for 2 min, then sprayed with ninhydrine solution (0.5% ninhydrine and 5 mM NaOH in ethanol/water 3:1 *v*/*v*).The R_f_ values were determined for the spots observed by illumination at 254 nm. Flash chromatography: Merck silica gel 60, 40–63 μm. All solvents were distilled prior to use. Reagents and materials were obtained from commercial suppliers and were used without purification. Elementary analysis data were determined with a CHN analyzer model 2400 (Perkin Elmer, Waltham, MA, USA). NMR spectra were obtained at room temperature with a DRX 500 instrument (Bruker, Rheinstetten, Germany). Chemical shifts are reported in ppm (δ scale), and coupling constants (*J*) in Hz. For the determination of multiplicities the J-modulated spin-echo pulse sequence was used. Mass spectra were recorded on a MAT TSQ 7000 instrument (Finnigan, Waltham, MA, USA) equipped with an electrospray ion source, using the following parameters: positive ionization mode, nebulizing gas N_2_ (3.45 bar), capillary temperature: 200 °C, capillary voltage: 4500 V and an acetonitrile–water 1:1 (*v*/*v*) mixture containing 0.1% trifluoroacetic acid as eluent. The samples were dissolved in acetonitrile and injected directly. The base peaks were usually the [M + H]^+^ signals along with far less abundant [2M + H]^+^ signals.

#### 3.1.1. General Procedure for Synthesis of 3′-*O*-Acetyl-*N*-acyl-protected-2′-deoxynucleosides **8a**–**d**

10 mmol of 5′-*O*-DMTr-*N*-acyl-protected-2′-deoxynucleoside **6a**, **6b**, **6c** or **6d** was dissolved in pyridine (50 mL) and Ac_2_O (10 mmol, 945 μL) was added. The reaction mixture was stirred at 0 °C for 4 h, evaporated in vacuo, redissolved in EtOAc (100 mL) and extracted with water (2 × 100 mL). The combined organic layers were dried over Na_2_SO_4_ and evaporated in vacuo. The resulting crude product **7a**, **7b**, **7c** or **7d** was directly used for the next step without further purifications.

10 mmol of 5′-*O*-(4,4′-dimethoxytrityl)-3′-*O*-acetyl-*N*-acyl-protected-2′-deoxynucleoside **7a**, **7b**, **7c** or **7d** was dissolved in nitromethane (20 mL) and a mixture of 1,1,1,3,3,3-hexafluoroisopropanol (20 mL, 190 mmol), boron trifluoride diethyl etherate (247 μL, 2 mmol) and triethylsilane (6 mL, 38 mmol) was added. The reaction mixture was stirred at room temperature overnight, then 10% aqueous NaHCO_3_ solution was added and evaporated in vacuo. The resulting crude product was purified by column chromatography with EtOAc as eluent to give the corresponding nucleoside **8a**, **8b**, **8c** or **8d**.

#### 3.1.2. General Procedure for Synthesis of 3′-*O*-Acetyl-5′-azido-*N*-acyl-protected-2′,5′-dideoxy-nucleosides **10a**–**c**

3′-*O*-Acetyl-*N*-acyl-protected-2′-deoxynucleoside **8a**, **8b**, **8c** or **8d** (1 mmol) was dissolved in pyridine (15 mL) and p-toluenesulfonyl chloride (285 mg, 1.5 mmol) was added. The reaction mixture was stirred at room temperature overnight, evaporated in vacuo, redissolved in EtOAc (100 mL) and extracted with 1% aqueous KHSO_4_ solution (2 × 100 mL). The combined organic layers were dried over Na_2_SO_4_ and evaporated in vacuo. The resulting crude product was purified by column chromatography with EtOAc as eluent to afford the tosylated nucleoside **9a**, **9b**, **9c** or **9d**.

3′-*O*-Acetyl-*N*-acyl-protected-2′-deoxy-5′-*O*-tosylnucleoside **9a**, **9b** or **9c** (1 mmol) was dissolved in dry DMF (20 mL) then sodium azide (195 mg, 3 mmol) and lithium bromide (261 mg, 3 mmol) were added. The reaction mixture was stirred at 50 °C for 6 h. The reaction mixture was evaporated in vacuo, redissolved in EtOAc and extracted with water (2 × 100 mL). The combined organic layers were dried over Na_2_SO_4_ and evaporated in vacuo. The resulting crude product was recrystallized from EtOAc to yield azidonucleoside **10a**, **10b** or **10c**.

*3′-O-Acetyl-5′-azido-6-N-benzoyl-2′,5′-dideoxyadenosine* (**10a**)**:** After purification, **10a** was obtained as a white solid (346 mg, 82%), m.p. 135–136 °C, *R*_f_ = 0.29 (ss A); ^1^H-NMR (CDCl_3_); δ [ppm] = 2.11 (s, 3H, 3′-OAc), 2.63 (d, 1H, *J* = 8.0 Hz) and 3.27 (m, 1H): 2′-H2, 3.61 (d, 1H, *J* = 11.5 Hz) and 3.76 (d, 1H, *J* = 12.0 Hz): 5′-H2, 4.27 (s, 1H), 5.39 (s, 1H), 6.54 (m, 1H): 1′-, 3′-, 4′-H, 7.55 (t, 2H, *J* = 2 × 7.0 Hz), 7.64 (d, 1H, *J* = 7.5 Hz), 8.06 (d, 2H, *J* = 7 Hz): benzoyl protons, 8.74 (s, 1H) and 8.79 (s, 1H): 2-H and 8-H, 11.23 (s, 1H, 6-NH); ^13^C-NMR (CDCl_3_); δ [ppm] = 20.7 (3′-OAc), 35.0 (C-2′), 51.5 (C-5′), 74.6, 82.9, 83.8, 125.9 (C-5), 128.3 (2C), 128.4 (2C), 132.4: benzoyl CHs, 133.2: benzoyl Cq, 143.3 (C-8), 150.4 (C-4), 151.9 (C-6), 152.3 (C-2), 165.5 (Bz-CO), 170.0 (Ac-CO); ESI-MS: 423 [M + H]+; Anal. Calcd for C_19_H_18_N_8_O_4_: C, 54.03; H, 4.30; N, 26.53. Found: C, 53.96; H, 4.47; N, 26.95.

*3′-O-Acetyl-5′-azido-5′-deoxythymidine* (**10b**): After purification, **10b** was obtained as a white solid (263 mg, 85%), m.p. 115–116 °C, *R*_f_ = 0.65 (ss A); ^1^H-NMR (CDCl_3_); δ [ppm] = 1.57 (s, 3H, 5-CH_3_), 1.83 (s, 3H, 3′-OAc), 2.02 and 2.27 (2 × m, 2 × 1H, 2′-H_2_), 3.40 (m, 2H), 3.86 (d, 1H, *J* = 2.0 Hz), 4.91 (d, 1H, *J* = 3.5 Hz), 5.95 (t, 1H, *J* = 10.0 Hz, *J* = 5.0 Hz): 1′-, 3′-, 4′-H, 5′-H2, 7.33 (s, 1H, 6-H), 11.16 (s, 1H, 3-NH); ^13^C-NMR (CDCl_3_); δ [ppm] = 12.0 (5-CH_3_), 20.7 (3′-OAc), 35.0 (C-2′), 51.6 (C-5′), 74.1, 81.9, 83.9, 109.9 (C-5), 135.9 (C-6), 150.4, 163.6, 170.0 (Ac-CO); ESI-MS: 310 [M + H]^+^; Anal. Calcd for C_12_H_15_N_5_O_5_: C, 46.60; H, 4.89; N, 22.64. Found: C, 46.82; H, 5.02; N, 22.83.

*3′-O-Acetyl-5′-azido-4-N-benzoyl-2′,5′-dideoxycytidine* (**10c**): After purification, **10c** was obtained as a white solid (302 mg, 76%), m.p. 146–147 °C, *R*_f_ = 0.32 (ss A); ^1^H-NMR (CDCl_3_); δ [ppm] = 2.11 (s, 3H, 3′-OAc), 2.53 (m, 2H, 2′-H_2_), 3.77 (dd, 2H, *J* = 9.5 Hz, *J* = 12.0 Hz, 5′-H_2_), 4.25 (s, 1H), 5.21 (s, 1H), 6.24 (d, 1H, *J* = 4.5 Hz): 1′-, 3′-, 4′-H, 7.42 (s, 1H, 5′-H), 7.54 (d, 2H, *J* = 5.5 Hz), 7.65 (d, 1H, *J* = 5.5 Hz), 8.04 (m, 2H): benzoyl protons, 8.24 (s, 1H, 6’-H), 11.34 (s, 1H, 4′-NH); ^13^C-NMR (CDCl_3_); δ [ppm] = 20.7 (3′-OAc), 36.6 (C-2′), 51.6 (C-5′), 74.3, 82.7, 86.5, 96.6 (C-5), 128.3 (2C), 128.4 (2C), 132.7: benzoyl CH-s, 133.1: benzoyl Cq, 145.2 (C-6), 154.1 (C-2), 163.6 (C-4), 165.1 (Bz-CO), 170.0 (Ac-CO); ESI-MS: 399 [M + H]^+^; Anal. Calcd for C_18_H_18_N_6_O_5_: C, 54.27; H, 4.55; N, 21.10. Found: C, 54.18; H, 4.63; N, 19.85.

#### 3.1.3. General Procedure for Click Reactions: Preparation of **12a**–**c**

Azidonucleoside **10a**, **10b** or **10c** (0.15 mmol) was dissolved in toluene (10 mL) or THF (10mL), CuI (0.225 mmol, 42.75 mg, 1.5 equiv.), DIPEA (78 μL, 0.45 mmol, 3 equiv.) and 3-*O*-propargyl-13α-estrone **11** [20] (0.165 mmol, 1.1 equiv.) were added to the reaction mixture and it was stirred for overnight at 50 °C. Then the mixture was evaporated in vacuo, the resulting crude product was purified by column chromatography with EtOAc as eluent to yield protected conjugate **12a**, **12b** or **12c**.

*3-{[1-(3′-O-Acetyl-6-N-benzoyl-2′,5′-dideoxyadenosine-5′-yl)-1H-1,2,3-triazole-4-yl]methyl-oxy}-13α-estra-1,3,5(10)-trien-17-one* (**12a**): After purification, protected conjugate **12a** was obtained as a white solid (78 mg, 68%), m.p. 141–143 °C, *R*_f_ = 0.55 (ss B); ^1^H-NMR (CDCl_3_); δ [ppm] = 1.00 (s, 3H, 18-CH_3_), 1.89 (s, 3H, 3′-OAc), 2.27 (dd, 2H, *J* = 10.5 Hz, *J* = 12.5 Hz, 2′-H_2_), 2.54 (d, 1H, *J* = 11.0 Hz) and 2.82 (d, 1H, *J* = 11.5 Hz): 6-H_2_, 2.55 (m, 1H), 4.45 (m, 1H), 4.84 (dd, 2H, *J* = 13.5 Hz, *J* = 14.0 Hz), 5.54 (s, 1H): 1′-, 3′-, 4′-H, 5′-H_2_, 5.04 (s, 2H, OCH_2_), 6.42 (s, 1H, 4-H), 6.61 (d, 1H, *J* = 6.0 Hz, 2-H) 6.69 (d, 1H, *J* = 8.5 Hz, 1-H), 7.06 (s, 1H), 7.46 (s, 3H), 7.55 (s, 1H), 8.00 (s, 2H): benzoyl protons, 2”-, 8”-H, 7.59 (s, 1H, HC=C), 8.72 (s, 1H, 6”-NH); ^13^C-NMR (CDCl_3_); δ [ppm] = 20.7 (C-18), 20.8, 20.9 (3′-OAc), 24.9 (2C), 30.2, 31.9, 33.3, 36.1 (C-2′), 41.2 (2C, C-8 and C-9), 49.1 (C-14), 50.0 (C-13), 51.4 (C-5′), 61.6 (OCH_2_), 74.4, 83.1, 85.3, 112.3 (C-2), 114.3 (C-4), 125.3 (HC=C), 123.8 (C-5”), 126.7 (C-1), 128.1 (2C). 128.6 (2C), 132.8: benzoyl CH-s, 132.4 (C-10), 135.7: benzoyl Cq, 138.0 (C-5), 139.5 (C-8”), 144.1 (HC=C), 147.1, 151.4, 155.9 (C-3), 153.4, 165.2 (Bz-CO), 170.3 (Ac-CO), 221.7 (C-17); ESI-MS: 731 [M + H]^+^; Anal. Calcd for C_40_H_42_N_8_O_6_: C, 65.74; H, 5.79; N, 15.33. Found: C, 65.67; H, 5.92; N, 15.46.

*3-{[1-(3′-O-Acetyl-5′-deoxythymidine-5′-yl)-1H-1,2,3-triazole-4-yl]methyloxy}-13α-estra-1,3,5(10)-trien-17-one* (**12b**): After purification, protected conjugate **12b** was obtained as a white solid (74 mg, 76%), m.p. 189–191 °C, *R*_f_ = 0.5 (ss A); ^1^H-NMR (CDCl_3_); δ [ppm] = 1.03 (s, 3H, 18-CH_3_), 1.24 (s, 2H), 1.87 (s, 3H, 5”-CH_3_), 2.09 (s, 3H, 3′-OAc), 2.20 (d, 2H, *J* = 11.0 Hz, 2′-H_2_), 2.78 (m, 2H, 6-H_2_), 4.29 (m, 1H), 4.84 (dd, 2H, *J* = 9.5 Hz, *J* = 11.5 Hz), 5.29 (s, 1H), 6.16 (m, 1H): 1′-, 3′-, 4′-H, 5′-H_2_, 5.17 (s, 2H, OCH_2_), 6.64 (s, 1H, 4-H), 6.72 (d, 1H, *J* = 6.0 Hz, 2-H), 6.90 (s, 1H, 6”-H), 7.14 (d, 1H, *J* = 8.5 Hz, 1-H), 7.79 (s, 1H, HC=C), 9.46 (s, 1H, 3”-NH); ^13^C-NMR (CDCl_3_); δ [ppm] =12.4 (5”-CH_3_), 20.8 (3′-OAc), 21.0, 25.0 (C-18), 28.2 (2C), 30.3, 32.0, 33.4, 36.0 (C-2′), 41.3 (2C, C-8 and C-9), 49.2 (C-14), 50.1 (C-13), 51.6 (C-5′), 61.6 (OCH_2_), 74.3, 82.0, 85.3, 111.8 (C-5”), 112.4 (C-2), 114.4 (C-4), 124.8 (HC=C), 126.9 (C-1), 132.7 (C-10), 135.6 (C-6”), 138.2 (C-5), 144.2 (HC=C), 150.3, 163.6, 155.8 (C-3), 170.6 (Ac-CO), 218.8 (C-17); ESI-MS: 618 [M + H]^+^; Anal. Calcd for C_33_H_39_N_5_O_7_: C, 64.17; H, 6.37; N, 11.34. Found: C, 64.25; H, 6.52; N, 11.49.

*3-{[1-(3′-O-Acetyl-4-N-benzoyl-2′,5′-dideoxycytidine-5′-yl)-1H-1,2,3-triazole-4-yl]methyl-oxy}-13α-estra-1,3,5(10)-trien-17-one* (**12c**): After purification, protected conjugate **12c** was obtained as a white solid (67 mg, 61%), m.p. 170 °C (dec.), *R*_f_ = 0.47 (ss B); ^1^H-NMR (CDCl_3_); δ [ppm] = 1.04 (s, 3H, 18-CH_3_), 2.13 (s, 3H, 3′-OAc), 2.63 (d, 2H, *J* = 9.0 Hz, 2′-H_2_), 2.78 (m, 2H, 6-H_2_), 4.43 (m, 1H), 4.82 (m, 2H), 5.24–5.26 (overlapping m, 2H): 1′-, 3′-, 4′-H, 5′-H_2_, 5.17 (s, 2H, OCH_2_), 6.68 (s, 1H, 4-H), 6.76 (d, 1H, *J* = 6.0 Hz, 2-H) 7.15 (d, 1H, *J* = 8.5 Hz, 1-H), 7.53 (t, 3H, *J* = 7.0 Hz), 7.64 (t, 2H *J* = 14.5 Hz): benzoyl protons, 7.73 (s, 1H, HC=C), 7.94 (s, 1H, 6”-H), 8.04 (s, 1H, 4”-NH); ^13^C-NMR (CDCl_3_); δ [ppm] = 20.8 (C-18), 21.0, 25.1 (3′-OCH_3_), 28.2 (2C), 29.6, 30.3, 32.0, 33.4, 37.5 (C-2′), 41.4 (2C, C-8 and C-9), 49.2 (C-14), 50.1 (C-13), 51.4 (C-5′), 56.3, 62.8 (OCH_2_), 74.1, 84.0, 97.3 (C-5”), 112.5 (C-2), 114.5 (C-4), 123.9 (C-1), 127.0 (HC=C), 129.2 (2C). 129.5 (2C), 132.6: benzoyl CH-s, 132.9 (C-10), 137.6: benzoyl Cq, 138.3 (C-5), 143.5 (HC=C), 156.0 (C-3), 157.5 (C-2”), 162.7 (C-4”), 170.6 (Bz-CO), 171.2 (Ac-CO), 220.5 (C-17); ESI-MS: 707 [M + H]^+^; Anal. Calcd for C_39_H_42_N_6_O_7_: C, 66.27; H, 5.99; N, 11.89. Found: C, 66.32; H, 6.13; N, 12.10.

#### 3.1.4. General Procedure for Deprotection of Conjugates **13a**–**c**

Protected conjugate **12a**, **12b** or **12c** (0.1 mmol) was deacylated by dissolving in 4 M NH_3_ in MeOH (5 mL), stirring the mixture overnight at room temperature and then the mixture was evaporated in vacuo and the resulting crude product was purified by column chromatography with EtOAc/acetonitrile (9:1) as eluent to afford unprotected conjugate **13a**, **13b** or **13c**.

*3-{[1-(2′,5′-Dideoxyadenosine-5′-yl)-1H-1,2,3-triazole-4-yl]methyloxy}-13α-estra-1,3,5(10)-trien-17-one* (**13a**): After purification, conjugate **13a** was obtained as a white solid (50 mg, 87%), m.p. 160 °C (dec.), *R*_f_ = 0.33 (ss B); ^1^H-NMR (DMSO-*d*_6_); δ [ppm] = 0.96 (s, 3H, 18-CH_3_), 2.71 (m, 2H, 2′-H_2_), 2.83 (m, 2H, 6-H_2_), 4.21 (s, 1H), 4.51 (s, 1H), 4.68-4.77 (overlapping m, 2H), 4.99 (dd, 2H *J* = 10.0 Hz, *J* = 12.0 Hz), 5.60 (s, 1H): 1′-, 3′-, 4′-H, 5′-H_2_, OCH_2_, 6.63 (s, 1H, 4-H), 6.70 (d, 1H, *J* = 8.0 Hz, 2-H) 7.12 (d, 1H, *J* = 8.5 Hz, 1-H), 7.47 (s, 2H, 6”-NH), 7.47 (s, 1H, HC=C), 8.21 (s, 1H), 8.32 (s, 1H): 2”-H and 8”-H; ^13^C-NMR (DMSO-*d*_6_); δ [ppm] = 20.5, 24.6 (C-18), 27.7, 28.0, 29.7, 31.6, 32.9, 38.0 (C-2′), 40.8 (2C, C-8 and C-9), 48.5 (C-14), 49.4 (C-13), 51.5 (C-5′), 60.8 (OCH_2_), 71.1, 83.8, 84.9, 112.4 (C-2), 114.1 (C-4), 119.1, 125.0 (HC=C), 126.7 (C-1), 132.0 (C-10), 137.9 (C-5), 140.1, 142.8 (HC=C), 150.4, 151.9, 155.6 (C-6”), 155.8 (C-3), 220.7 (C-17); ESI-MS: 585 [M + H]^+^; Anal. Calcd for C_31_H_36_N_8_O_5_: C, 63.68; H, 6.21; N, 19.17. Found: C, 63.84; H, 6.35; N, 19.38.

*3-{[1-(5′-Deoxythymidine-5′-yl)-1H-1,2,3-triazole-4-yl]methyloxy}-13α-estra-1,3,5(10)-trien-17-one* (**13b**): After purification, conjugate **13b** was obtained as a white solid (51 mg, 89%), m.p. 211–212 °C, *R*_f_ = 0.63 (ss B); ^1^H-NMR (DMSO-*d*_6_); δ [ppm] = 0.97 (s, 3H, 18-CH_3_), 1.77 (s, 3H, 5”-CH_3_), 1.88 (m, 1H) and 2.30 (m, 1H): 2′-H_2_, 2.74 (d, 2H, *J* = 3.0 Hz, 6-H_2_), 4.28 (m, 1H), 4.63 (m, 1H) and 4.71 (m, 1H): 5′-H_2_, 5.01 (s, 2H, OCH_2_), 4.09 (d, 1H, *J* = 3.0 Hz), 5.50 (s, 1H), 6.16 (t, 1H, *J* = 5.0 Hz, *J* = 6.0 Hz): 1′-, 3′-, 4′-H, 6.69 (s, 1H, 4-H), 6.76 (d, 1H, *J* = 8.5 Hz, 2-H), 7.16 (d, 1H, *J* = 7.5 Hz, 1-H), 7.33 (s, 1H, HC=C), 8.16 (s, 1H, 3”-NH); ^13^C-NMR (DMSO-*d*_6_); δ [ppm] = 12.0 (5”-CH_3_), 20.4, 24.5 (C-18), 27.6, 27.9, 29.7, 31.5, 32.5, 37.8 (C-2′), 40.7 (2C, C-8 and C-9), 48.4 (C-14), 49.4 (C-13), 51.1 (C-5′), 60.8 (OCH_2_), 70.7 (C-3′), 83.8, 83.9, 109.8 (C-5”), 112.3 (C-2), 114.1 (C-4), 125.0 (HC=C), 126.6 (C-1), 131.9 (C-10), 135.9 (C-6”), 137.8 (C-5), 142.9 (HC=C), 150.3, 155.7 (C-3), 163.5, 220.5 (C-17); ESI-MS: 576 [M + H]^+^; Anal. Calcd for C_31_H_37_N_5_O_6_: C, 64.68; H, 6.48; N, 12.17. Found: C, 64.53; H, 6.62; N, 12.35.

*3-{[1-(2′,5′-Dideoxycytidine-5′-yl)-1H-1,2,3-triazole-4-yl]methyloxy}-13α-estra-1,3,5(10)-trien-17-one* (**13c**): After purification, conjugate **13c** was obtained as a white solid (45 mg, 81%), m.p. 170 °C (dec.), *R*_f_ = 0.41 (ss C); ^1^H-NMR (DMSO-*d*_6_); δ [ppm] = 0.96 (s, 3H, 18-CH_3_), 2.29 (m, 2H, 2′-H_2_), 2.74 (m, 2H, 6-H_2_), 3.75 (m, 1H), 4.21 (m, 2H), 4.62-4.72 (overlapping m, 2H): 1′-, 3′-, 4′-H, 5′-H_2_, 5.06 (s, 2H, OCH_2_), 6.70 (s, 1H, 4-H), 6.75 (d, 1H, *J* = 6.5 Hz, 2-H) 7.15 (d, 1H, *J* = 8.0 Hz, 1-H), 7.29 (s, 1H, HC=C), 8.04 (d, 1H, *J* = 6.5 Hz, 6”-H), 8.19 (s, 1H, 4”-NH2); ^13^C-NMR (DMSO-*d*_6_); δ [ppm] = 24.6 (C-18), 27.8, 28.1, 29.8, 31.7, 33.0, 36.1 (C-2′), 40.8 (2C, C-8 and C-9), 43.4, 48.6 (C-14), 50.5 (C-13), 51.5 (C-5′), 61.3 (OCH_2_), 84.1, 84.9, 87.3, 94.5 (C-5”), 112.5 (C-2), 114.2 (C-4), 125.2 (C-1), 126.8 (HC=C), 132.1 (C-10), 137.9 (C-5), 141.1 (C-6”), 142.9 (HC=C), 155.0 (C-3), 155.2 (C-2”), 165.6 (C-4”), 220.7 (C-17); ESI-MS: 561 [M + H]^+^; Anal. Calcd for C_30_H_36_N_6_O_5_: C, 64.27; H, 6.47; N, 14.99. Found: C, 64.42; H, 6.34; N, 15.18.

### 3.2. Cell Cultures and Antiproliferative Assays

The growth-inhibitory effects of the conjugates **12a**–**c** and **13a**–**c** were tested in vitro by means of the MTT assay on a panel of malignant human cell lines of gynecological origin including HeLa, MCF-7 and A2780 cells, isolated from cervical, breast and ovarian cancers. The cell lines were obtained from the European Collection of Cell Cultures (Salisbury, UK). The cells were maintained in minimal essential medium supplemented with 10% fetal bovine serum (FBS), 1% nonessential aminoacids and an antibiotic-antimycotic mixture (AAM). All media and supplements were obtained from Lonza Ltd. (Basel, Switzerland). All chemicals, if otherwise not specified, were purchased from Sigma-Aldrich Ltd. (Budapest, Hungary). All cell lines were grown in a humidified atmosphere of 5% CO_2_ at 37 °C. For pharmacological investigations, 10 mM stock solutions of the tested conjugates **12a**–**c** and **13a**–**c** and cisplatin controls were prepared in dimethyl sulfoxide (DMSO). The highest applied DMSO concentration of the medium (0.3%) did not exert any substantial effect on the determined cellular functions. All cell types were seeded into 96-well plates at a density of 5000 cells/well and allowed to stand overnight under cell-culturing conditions, then the medium containing the tested compounds (**12a**–**c** and **13a**–**c** and cisplatin controls) were added. After a 72 h incubation with increasing concentrations (0.03−30 µM) of the compounds (**12a**–**c** and **13a**–**c** and cisplatin controls), viability was determined by the addition of 20 µL MTT solution (5 mg/mL) for 4 h. The precipitated formazan crystals were solubilized in DMSO and the absorbance values were determined at 545 nm with an ELISA reader [62] utilizing untreated cells as controls. Two independent experiments were performed with 5 parallel wells and cisplatin, a clinically applied anticancer agent, was used as reference compound. For the most effective conjugates (**12a**, **12c** and **13a**) sigmoidal dose-response curves were fitted to the measured data in order to determine the IC_50_ values by means of Graphpad Prism 4.0 (Graphpad Software; San Diego, CA, USA).

### 3.3. Determination of 17β-HSD1 Inhibition

The inhibitory effects exerted on 17β-HSD1 activity by the newly synthesized conjugates were determined via an in vitro radiosubstrate incubation method described in detail earlier [22,63]. Conversion of estrone to 17β-estradiol was measured in the presence of NADPH cofactor and microsomes of human termed placenta served as enzyme source. Relative conversions in the presence of 10 μM test compounds were measured. IC_50_ value (the inhibitor concentration that decreases the enzyme activity to 50%) was determined for **12b**. A reference IC_50_ value was determined earlier for unlabelled estrone and that was 0.63 ± 0.11 μM.

## 4. Conclusions

Triazolyl nucleoside conjugates of 13α-estrone have been synthesized succesfully in order to investigate their antiproliferative and 17β-HSD1 enzyme inhibitory activity. The CuAAC reaction conditions have been optimized for the coupling of the protected azidonucleosides with 3-*O*-propargyl-13α-estrone. The biological assays showed lower antiproliferative and 17β-HSD1 enzyme inhibitory activities for the conjugates compared to our earlier synthesized 3-*O*-[(1-benzyl-1*H*-1,2,3-triazol-4-yl)methyl]-13α-estrone (**1**) which is probably due to the steric effect and the polar behavior of the inserted nucleoside units. The protected, cytidine–13α-estrone conjugate **12c** showed the best antiproliferative activity with IC_50_ values of 9.0–10.4 µM and the unprotected, thymidine–13α-estrone conjugate **13b** displayed the best enzyme inhibitory potential (IC_50_ = 19 µM). 13α-Estrone lost its 17β-HSD1 inhibitory activity upon attaching the nucleoside moiety to its 3-OH group by a triazolylmethyl linker. The antiproliferative activity was also decreased by changing the benzyl group for the nucleoside unit in our potent model compound **1**. Concerning that the most active 17β-HSD1 inhibitor was different from the best cell growth-inhibitor in this series, it can be stated, that the two biological effects should be attributed to other mechanisms. From the biological results, it seems that neither the improvement in the activity nor the improvement in the selectivity was demonstrated for the synthesized nucleoside–13α-estrone conjugates.

## Figures and Tables

**Table 1:** Optimization of CuAAC conjugation reaction of 3′-*O*-acetyl-5′-azido-2′,5′-dideoxy- nucleosides and 3-*O*-propargyl-13α-estrone.

	Conditions and Yields of CuAAC Reaction					
	Ref. [32] ^1^		Refs. [36,38,61] ^2^		This work ^3^	
Product Code	Product Yield (%)	Recovered Nucleoside (%) ^4^	Product Yield (%)	Recovered Nucleoside (%) ^4^	Product Yield (%)	Recovered Nucleoside (%) ^5^
**12a**	0	93	18–22	63–68	68	-
**12b**	0	92	23–28	64–70	76	-
**12c**	0	>95	8–11	78–82	61	-

^1^ 1 mol % CuSO_4_, 5% (*m*/*v*) aq. sodium ascorbate, water: *tert*-butanol 2:1 (*v*/*v*), r.t., 8 h; ^2^ 0.01–0.2 equiv. CuI, 0.2 equiv. Ph_3_P, 0.2 equiv. DIPEA, toluene (**12a**, **12b**) or THF (**12c**), 50 °C, 16–72 h; ^3^ 1.5 equiv. CuI, 3 equiv. DIPEA, toluene (**12a**, **12b**) or THF (**12c**), 50 °C, 16 h; ^4^ Determined using TLC densitometry of the UV-active spots; ^5^ Not determined.

**Table 2:** Antiproliferative properties of the synthesized compounds. Mean value from two independent determinations with five parallel wells; standard deviation <15%.

Structure	Compd Code or Name [ref.]	Conc. (µM)	Inhibition (%) ± SEM [Calculated IC_50_, µM] ^1^		
			A2780	HeLa	MCF-7
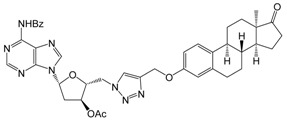	**12a**	10	39.4 ± 2.4	- ^2^	- ^2^
		30	70.2 ± 1.6	55.5 ± 0.6	49.6 ± 1.4
			[10.9]	[16.3]	[>30] ^3^
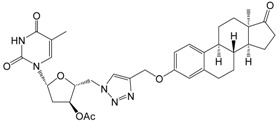	**12b**	10	29.8 ± 0.2	-	29.2 ± 2.9
		30	35.1 ± 2.7	-	26.7 ± 2.0
			[>30]	[>30]	[>30]
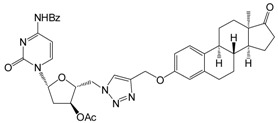	**12c**	10	63.2 ± 1.5	53.5 ± 1.0	47.4 ± 2.4
		30	66.6 ± 1.6	61.9 ± 1.2	57.3 ± 1.8
			[9.0]	[9.0]	[10.4]
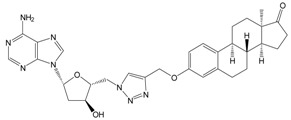	**13a**	10	-	26.6 ± 1.8	-
		30	41.9 ± 1.7	60.1 ± 0.7	36.6 ± 1.0
			[>30]	[23.5]	[>30]
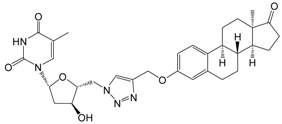	**13b**	10	-	25.9 ± 0.8	-
		30	32.4 ± 2.0	38.2 ± 2.1	-
			[>30]	[>30]	[>30]
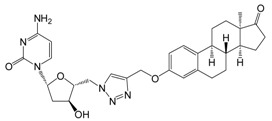	**13c**	10	31.8 ± 2.5	41.4 ± 1.5	-
		30	31.6 ± 3.6	46.1 ± 2.6	26.4 ± 2.2
			[>30]	[>30]	[>30]
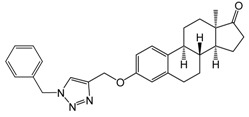	**1** [20]	10	77.5 ± 0.4	90.9 ± 0.3	85.8 ± 1.3
		30	78.4 ± 0.9	93.3 ± 0.2	85.0 ± 0.2
			[0.5]	[0.9]	[0.6]
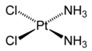	cisplatin	10	83.6 ± 1.2	42.6 ± 2.3	66.9 ± 1.8
		30	95.0 ± 0.3	99.9 ± 0.3	96.8 ± 0.4
			[1.3]	[12.4]	[5.8]

^1^ Mean value from two independent determinations with five parallel wells; standard deviation <15%; ^2^ Inhibition values <20% are not presented. ^3^ IC_50_ values > 30 µM are not calculated.

**Table 3:** Inhibition results on 17β-HSD1. Relative conversions (control incubation with no inhibition is 100%) measured in the presence of 10 μM of the compound tested. IC_50_: The inhibitor concentration that decreases the enzyme activity to 50%. SD: standard deviation (for relative conversion n = 3).

Structure	Compd Code	Relative conversion ± SD at 10 µM (%) [IC_50_ ± SD (µM) NADPH]
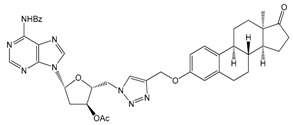	**12a**	90 ± 13
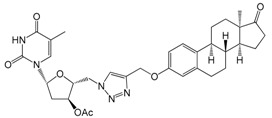	**12b**	89 ± 7
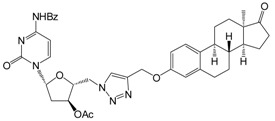	**12c**	98 ± 14
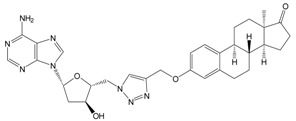	**13a**	113 ± 6
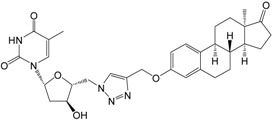	**13b**	65 ± 3 [IC_50_ = 19 ± 10]
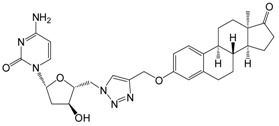	**13c**	91 ± 4
